# Race implications of COVID-19 in Utah: a population-based cohort study

**DOI:** 10.21203/rs.3.rs-10059652/v1

**Published:** 2026-09-08

**Authors:** Natalia Arizmendez, Megan A. Cahn, Samuel Yousefzai, Zhe David Yu, Kimberley Shoaf, Sharon L. Talboys, Daniel B. Knox, Chun-Pin Esther Chang, Mia Hashibe

**Affiliations:** Intermountain Healthcare; Legacy Health; Legacy Health; Huntsman Cancer Institute; University of Utah School of Medicine; University of Utah School of Medicine; Intermountain Healthcare; University of Utah School of Medicine; UCLA Fielding School of Public Heath

## Abstract

**Background:**

The COVID-19 pandemic disproportionately affected marginalized populations in the United States. We characterize statewide COVID-19 health outcomes by race and ethnicity.

**Methods:**

We conducted a retrospective analysis of individuals alive on January 1, 2020 with known sex, race, and ethnicity data utilizing the Utah Population Database. COVID-19 health outcomes were identified by diagnostic codes. Ethnically diverse individuals were matched to non-Hispanic White (NHW) individuals by age and sex. Demographic characteristics were compared using Chi-square test. Conditional logistic regression models were used to estimate odds ratios (OR) for COVID-19 related outcomes with adjustment for potential confounders.

**Results:**

Among 1, 230, 862 individuals, 50% were NHW, 37% Hispanic/Latino, 2.6% Asian, 1.9% Black/ African American, 1.1% Native Hawaiian/ Pacific Islander (NHPI), 0.85% America Indian/ Alaskan Native (AIAN), and 6.6% multiracial. NHPI were the youngest, median age of 26. NHPIs had over a 4-fold increase in hospitalization and 11.28-fold higher odds of COVID-19-specific death compared to NHW. AIAN had more than 2-fold higher odds of hospitalization, ICU admission, death due to COVID-19 compared to NHW.

**Conclusions:**

Significant racioethnic disparities in COVID-19 health outcomes were observed in NHPI and AIAN populations and underscore the need for targeted public health strategies to mitigate disparities.

## BACKGROUND

The COVID-19 pandemic was one of the worst 21st-century worldwide health crises, ushering in widespread public health as well as socioeconomic consequences. It was estimated by 2020 that COVID-19 was responsible for over 3 million excess deaths globally, and its effects were disproportionately felt among various racial and ethnic populations [1-3]. Several studies in the United States (US) have found striking racial disparities in infection, hospitalization, and death [2, 3]. These have been especially pronounced among Black/African American (AA), Hispanic/ Latino, and Native American/ Alaskan Native (AIAN) communities, who have suffered disproportionately high rates of severe illness and death relative to their non-Hispanic White (NHW) counterparts. Nevertheless, existing literature is constrained by data quality limitations, insufficient control for confounding variables, and a lack of comprehensive state-level analyses.

Systemic racism has historically loomed large over health outcomes, intricately influencing differences in access to health care, economic security, housing, and job opportunities [4-7]. Previous underlying social determinants of health, such as employment, housing, and underlying chronic diseases, have also contributed significantly to the worsening of COVID-19 disparities. Minoritized communities are overrepresented in occupations that expose them to greater risks of infection, such as retail workers, food preparers, health care support, and construction [2, 5, 8, 9]. These occupations most frequently involve in-person labor with little to no remote work possibilities, which also elevates the risk of viral transmission. Also, racial and ethnic minoritized communities are more likely to reside in close living conditions, where multi-generational households and limited living quarters greatly increase the risk of COVID-19 exposure and infection [10].

Aside from exposure risk, differences in underlying health conditions have also affected COVID-19 severity among minoritized racial groups. Chronic diseases such as diabetes, hypertension, and obesity are prevalent among people of color, a predicament based on historical and current limitations to healthcare, healthy food, and recreational activities [6, 7]. These conditions have been linked with worse outcomes in COVID-19 infections [11, 12]. In addition, access to COVID-19 prevention measures, including vaccinations and early treatment, has not been equitable between racial and socioeconomic lines, which further exacerbates the gaps in disease severity and death. The convergence of these factors highlights the necessity for a combined and evidence-driven examination, aimed at an enhanced comprehension of the mechanisms underlying these racial disparities and guiding effective public health initiatives.

Despite an increasing body of literature on racial inequalities during the pandemic, stronger studies that adjust for key confounders and provide nuanced state-level analyses remain necessary. Many prior studies have been unable to successfully control co-morbidities and other socioeconomic factors, which have limited their ability to determine the extent to which COVID-19 outcome disparities are attributable to underlying health conditions versus structural inequities [2, 13, 14]. To address these limitations, our analysis provides one of the first comprehensive, state-level analyses of racial and ethnic disparities in COVID-19 outcomes in Utah. Utilizing the Utah Population Database, we leverage data from several large healthcare systems to provide a detailed analysis of racial and ethnic inequities in hospitalization with COVID-19 diagnosis, Intensive Care Unit (ICU) admission with COVID-19 diagnosis, and mortality. The main aim of the present study is to assess the degree of racial and ethnic differences in COVID-19 health outcomes in Utah and assess the association of pre-existing comorbidities.

The aim of this study is to compare COVID-19 health outcomes with and without comorbidity adjustment to quantify the degree to which health disparities can be attributed to underlying health status, to estimate independent associations among patients with similar clinical risk profiles and distinguish structural and systemic contributions. Utilizing rigorous methods, state-level data, and a lengthy study period that follows the course of the pandemic, our study aims to provide novel insights into the persistent racial inequities that characterized COVID-19 outcomes in Utah.

## MATERIALS AND METHODS

The study was approved by the Institutional Review Board at the University of Utah and the Resource for Genetic and Epidemiologic Research (oversight committee for the Utah Population Database). This study was conducted in accordance with the ethical standards of the responsible committee on human experimentation (institutional or regional) and with the Helsinki Declaration of 1975. It was approved by our internal institutional review board on 12/13/2022. Given the retrospective nature of the study, ethics approval and consent to participate is not applicable as the requirement for informed consent was waived by internal institutional review, (IRB 146514).

### Data source

We used the Utah Population Database (UPDB) to conduct a population-based cohort study. UPDB links data from the All-Payer Claims Data (APCD), statewide healthcare facility data, electronic medical records (EMR), voter registration, residential histories, and birth and death certificates for the population of the state of Utah. Records from the hospital EMR were obtained from the two major healthcare systems in Utah: the University of Utah Health and Intermountain Health [15]. The statewide healthcare facility data includes ambulatory surgery, emergency department encounter dates, and encounter records for inpatient discharge, regardless of payer source (uninsured, private insurance, Medicaid, Medicare, and Public Employee Health Plans (PEHPs)) [10].

### Study population

The study included Utah residents alive on January 1, 2020, with known sex, race, and ethnicity. We identified 615,431 individuals within racial and ethnic groups and matched them to NHW individuals by age and sex with a one-to-one ratio.

### Study variables

The baseline date was defined as January 1, 2020. Race is a proxy for the experience of racism and racialization in healthcare systems and not a biological category. Therefore, we use predefined racial, ethnic, and sex categories described by the UPDB. The zip code within two years of the index date was used to identify socioeconomic status (SES), which includes county-level income and county-level education. The first hospitalization and ICU admission events occurring between February 2020 and September 2023 were identified. Hospitalizations were defined using procedure codes 99221, 99222, 99223, 99231, 99232, 99233, 99234, 99235, 99236, 99238, and 99239, or an inpatient flag from statewide healthcare facility data, with a COVID-19 diagnosis (ICD-10 codes: B97.29, U07.1, U08, U08.9, U10, U10.9, Z86.16, U09, U09.9). ICU admissions were identified using procedure codes 99291 and 99292 with a COVID-19 diagnosis. All-cause death and COVID-19-specific death were identified from the death certificates. Deaths with U07.1 listed as the primary cause of death were classified as COVID-19–specific death. The baseline Charlson Comorbidity Index (CCI) was calculated using data from one year prior to the index date up to the index date [16].

### Statistical analysis

Demographic characteristics between individuals with varying racial and ethnic groups were compared using the Chi-square (χ^2^) test. Conditional logistic regression models were used to estimate odds ratios (OR) and 95% confidence intervals (CI) for the odds of COVID-19 related outcomes with adjustment for potential confounders, including race, ethnicity, birthplace, baseline CCI, SES, and obesity. Descriptive statistics were used to summarize demographics and clinical characteristics; frequency and percentage for categorical variables and median and interquartile ranges (IQR) for continuous variables. Potential confounding factors were chosen a priori based on the three properties of confounders determined by a causal model, a directed acyclic graph (DAG) [7]. Three statistical models were used to assess for the relationship between COVID-19 outcomes and race/ethnicity adjusting for sex, age, and birthplace (Model 1), additionally adjusting for baseline CCI and SES (Model 2), and additionally adjusting for obesity (Model 3). Analyses were performed using SAS software (version 9.4, SAS Institute).

This work was funded by grants from the CDC OT21-2103 as part of the Reducing Health Disparities OT21 Grant through Utah Department of Health and Human Services. Research was supported by the NCRR grant, “Sharing Statewide Health Data for Genetic Research” (R01 RR021746, G. Mineau, PI) with additional support from the Utah Department of Health and Human Services and the University of Utah. Partial support for all datasets within the Utah Population Database was provided by the University of Utah Huntsman Cancer Institute and the Huntsman Cancer Institute Cancer Center Support grant, P30 CA2014 from the National Cancer Institute. The computational resources used were partially funded by the NIH Shared Instrumentation Grant 1S10OD021644-01A1.

## RESULTS

### Sample Characteristics

In our study, 615,431 individuals identified as NHW, 455,356 as Hispanic/Latino, 32,499 as Asian, 22, 903 as Black/AA, 13,115 Native Hawaiian/ Pacific Islander (NHPI), 10,424 as AIAN, and 81,134 identified as multiracial (MR). NHPI were the youngest with a median age of 26 [IQR, 8–40]. Asian individuals had a higher median age at baseline, 37 (IQR, 20–53) and NHW had a median age of 29 (IQR, 14–45). (Table 1). Minoritized groups were more likely to be born outside of the US, 26.1% of Hispanic/Latino, 57.8% of Asian, 23.6% of Black/AA, and 20.0% of NHPI.

Larger percentages of NHW (19.8%) and Asian (19.1%) Utah residents lived in counties with the lowest level of high school graduates (less than seven percent) compared to AIAN (6.1%) and Black/AA (11.3%) residents. Conversely, NHW (29.1%) and Asian (30.2%) residents were more likely than other racial and ethnic groups to live in a county with a median household income of $100,000 or more. In contrast, 63.6% of NHPI residents lived in counties with the lowest median household income of less than $60,000. NHW residents (87.3%), NHPI (87.7%) and Hispanic/Latino (87.2%) were more likely than other racial/ethnic groups to have a 0 score on the Charlson Comorbidity Index while MR Utah residents were more likely to have a score of 2+ (7.8%). Obesity at baseline was most common in Utahn NHPI (32.8%) than all other racial groups at baseline.

### Model 1: Adjusting for Sex, Age, and Birthplace

#### Hospitalization with COVID-19 Diagnosis.

Compared to NHW, the odds of hospitalization with COVID-19 diagnosis was greatest in NHPI (OR 4.34; 95% CI, 3.49, 5.39), AIAN (OR 2.20; 95% CI, 1.79, 2.69), Hispanic/Latino (OR 1.53; 95% CI, 1.47, 1.60), and MR individuals (OR = 2.03; 95% CI, 1.88, 2.19) (Table 2, Model 1). Asian communities exhibited lower likelihood of COVID-19 hospitalization (OR 0.74; 95% CI, 0.64, 0.84) compared to NHW. There was no statistically significant difference in the likelihood of hospitalization in Black/AA compared to NHWs (OR 1.11; 95% CI, 0.95, 1.31).

#### ICU Admission with COVID-19 Diagnosis.

Compared to NHW, the odds of ICU admission with COVID-19 diagnosis were greatest in NHPI (OR 4.60; 95% CI, 3.21, 6.60). Higher odds of ICU admission related to COVID-19 diagnosis were also higher in AIAN (OR 2.72; 95% CI, 1.98, 3.74), and MR individuals (OR 2.46; 95% CI, 2.16, 2.80), Hispanic/Latino (OR 1.58; 95% CI, 1.47, 1.69), (Table 2, Model 1). There were no significant differences in the ICU admission with COVID-19 diagnosis for Black/ AA or Asian individuals compared to NHW.

#### All-Cause Death.

A 4.7-fold increase in all-cause mortality was observed in NHPI compared to NHW (OR 4.70; 95% CI, 3.46–6.37). Higher likelihood of all-cause death was also increased in Hispanic (OR 1.37; 95% CI, 1.30, 1.45), Black/AA (OR 1.66; 95% CI, 1.35, 2.06), AIAN (OR 2.04; 95% CI, 1.59, 2.61), and MR (OR 1.89; 95% CI, 1.72, 2.07) individuals when compared to NHWs. There was no difference in all-cause death in Asian groups when compared to NHW.

#### COVID-19 Specific Death.

In comparison to NHW, NHPI experienced the greatest COVID-19 related mortality (OR 10.74; 95% CI, 3.85, 29.96). Increased odds of COVID-19 death were also increased in AIAN by nearly 7-fold and over 2-fold in MR and Hispanic/Latino groups when compared to NHW (Table 2, Model 1). There was no association with COVID-19 specific mortality in Black/AA and Asian groups.

### Models 2 and 3: Adjusting for Sex, Age, Birthplace, SES, and Comorbidities

We analyzed the association between race/ethnicity and COVID-19 health outcomes that were impacted by SES and comorbidities defined by CCI (Model 2) and the influence of obesity (Model 3). Compared to Model 1, nearly all significant differences remain. There was minimal change in the magnitude and no change in the direction of the associations between COVID-19 health outcomes across racial and ethnic groups when controlling for SES and comorbidities (Table 2, Model 2 and 3). NHPI consistently had highest adjusted odds of hospitalization with COVID-19, ICU admission with COVID-19, and all cause and COVID-19 related mortality. Similarly, the results remained consistent in adjusted analyses for obesity. Compared to NHW counterparts, NHPI individuals experienced attenuated but statistically significant higher likelihood of COVID-19 specific hospitalization (OR 3.39; 95% CI, 2.69, 4.25), ICU admission with COVID-19 diagnosis (OR 3.44; 95% CI, 2.35, 5.03), all-cause mortality (OR 4.10; 95% CI, 2.98, 5.65), and COVID-19 specific death (OR 10.16; 95% CI, 3.57, 28.91). In comparison to NHW, Asian groups experienced a protective or inverse association in other models adjusting for birthplace and social influence (SES and CCI). After adjusting for obesity, the association between hospitalization with COVID- 19 and Asian communities was no longer statistically significant (OR 0.86; 95% CI, 0.74–1.01). (Table 2, Model 3).

In adjusted models investigating COVID-19 outcomes and the interaction between sex and race/ethnicity, there was statistically significant higher odds of hospitalization with COVID-19 diagnosis in NHPI when compared to NHW. NHPI men experienced a 4.27-fold increase in hospitalization with a COVID-19 diagnosis (95%CI, 3.05, 5.98) and women experienced a 2.66-fold increase in hospitalization with a COVID-19 diagnosis (95% CI, 1.95, 3.64) compared to the respective sex among NHW, p = 0.03. A statistically significant increase odds of all-cause mortality was observed in AIAN men compared to NHW men (OR 2.42, 95%CI, 1.67, 3.50, p = 0.022). (Table 3). There were no significant differences in ICU admission with COVID-19 diagnosis or COVID-19 specific death.

To assess the effects of obesity on COVID-19 outcomes by race and ethnicity, we created a separate model with interaction terms between obesity and race/ethnicity (Table 4). Obese NHPI men had 2.97-fold higher odds of hospitalization with COVID-19 diagnosis compared to obese NHW men. Although non-obese NHPIs had lower odds of hospitalization with COVID-19 compared to obese NHPI, there was a 2.56-fold increase in hospitalization with COVID-19 diagnosis in non-obese NHPI compared to nonobese NHW. The interaction between obesity and NHPI was statistically significant (p = 0.025). In comparison to NHW, obese MR individuals had a 2.27-fold increase in ICU admission with COVID-19 diagnosis and non-obese MR individuals had a 1.68-fold increase in COVID-19 specific ICU admission, with effect modification by obesity, p interaction = 0.002. Among obese Black/AA there was no association with all-cause mortality (OR 1.00, 95%CI, 0.79, 1.34, p = 0.014). In contrast, non-obese Black/AA individuals experienced nearly 1.5 higher odds of all-cause mortality compared to NHW individuals (OR 1.46; 95% CI, 1.23, 1.73) with p for interaction = 0.014.

## DISCUSSION

The impacts of the COVID-19 pandemic on health outcomes in the US are still being extricated. COVID-19 increased the mortality across most marginalized populations [2, 8, 17]. Race is a proxy for the experience of racism in healthcare systems not a biological category. Many of the studies reviewing outcomes in COVID-19 report racial and ethnic inequities in large US cities yet there is a paucity of literature investigating geographically diverse areas such as Utah. Furthermore, patterns in underrepresented marginalized communities like MR, AIAN, and NHPI are not fully defined. This comprehensive review of the impact of COVID-19 on the communities in Utah reflects racial and ethnic disparities and highlights contextual differences in population composition and data completeness. This research adds to the broader literature by building a more detailed picture of racial and ethnic health disparities, guiding public health policy, and calling for systemic changes that ensure equitable access to healthcare and improved outcomes for all populations.

Consistent with nationwide findings, racial and ethnically diverse Utah residents experienced disproportionately worse COVID-19 health outcomes [2, 18-20]. We found strong associations with race and ethnicity, even after controlling factors associated with increased severity and worse COVID-19 outcomes [21]. Expansive reviews describe increase in all-cause mortality and excess death in pre-specified age groups [22]. A disproportionately higher all-cause mortality was observed in Utah Hispanic/Latino, Black/AA, AIAN, NHPI, and MR groups. The most pronounced burden of COVID-19 health outcomes among Utah population was observed in NHPI and AIAN. Interestingly, controlling for SES and comorbidities did not result in a significant reduction in mortality. Unpacking the disproportionate outcomes in COVID-19 diagnoses and mortality are complex. Marginalized populations continued to experience substantial health outcomes compared to NHW independent of SES, comorbidities, and obesity. These factors only partially explain differences in health outcomes related to COVID-19. There are sociological and systemic factors minoritized communities experience that are not fully captured by SES, such as occupational exposures, household living conditions, vaccine access and receipt, and hospital level variables [2, 5, 8, 9, 23, 24].

Black/AA, and Hispanic/Latino, AIAN, and MR groups historically have high risk occupations compared to NHW workers [25]. High risk occupations include increased risk of illness, work related injuries and fatality with less compensation and protected time for injury or illness [5, 8, 25]. Minoritized individuals suffer from historical laws that systematically exclude them from equal protections such as the policies that provided occupational safeguards to NHW employees excluded ethnically diverse employees [5]. The lack of minimum wage protection and lower compensation for high-risk occupations influences socioeconomic standing and educational attainment directly impacting disproportionate health outcomes and mortality [4, 5, 26]. Furthermore, hospitals serving minoritized communities consistently have worse health outcomes and lower performance [23, 27]. We did not account for hospital level factors which may influence outcomes given the rural geographic pattern of Utah and the strain on access to health care among AIAN populations throughout the region [28]. Consistent evidence describes high risk and age adjusted all-cause mortality in Hispanic/Latino, comparable to Utah Hispanic/Latino community [2]. NHPI and AIAN experienced the largest impact, particularly COVID-19 specific death. These groups along with other minoritized communities are disproportionately concentrated in the lowest county-level household income, similar to nationwide data [4]. Black/ AA comprise a relatively small proportion of the state’s demographic profile. This underrepresentation in Utah explains the statistical challenges and divergence from national trends in COVID-related outcomes. While we report similar racial and ethnic trends to national patterns, differences in racial and ethnic composition, methodology, limited representation of specific ethnic groups (AIAN, NHPI, Black/AA) make direct comparisons challenging. Utah Asians had a lower likelihood of hospitalization, ICU admission and mortality deviating from national trends [2, 29, 30]. Disaggregated Asian populations experience heterogenous health comes related to COVID-19. Limited by population size, we were unable to fully assess Asian subgroups which could partially explain differences in outcomes nationwide.

We have several limitations in our study. First, we report observed COVID-19 outcomes, thus it is possible that the true and excess mortality is higher as a result of limited COVID-19 test availability, varying COVID-19 related death definition and documentation, and those who may have died prior to seeking medical attention. SES was modeled at a county level and not individual level, which therefore may not accurately reflect an individual’s income level or household size. For example, among our cohort, AIAN reside in a disproportionately lower median household income yet lived in an area with the highest high school degree completion suggesting discordance and limitation in socioeconomic status. Vaccination data was not available and may influence the health outcomes amongst our population.

## CONCLUSION

These observations highlight the importance of both local demographic structure and comprehensive, disaggregated racial and ethnic data when investigating racial and ethnic disparities in health outcomes. Improved standardized race/ethnicity data collection and reporting are essential to accurately characterize the impact of COVID-19 on vulnerable communities. This necessary research allows for the development of correlation studies that guide policy development and implementation targeting health equity [22, 31].

## Figures and Tables

**Table 1:** The distribution of demographic characteristics in Utah, by race and ethnicity

	Non- Hispanic White (n = 615,431)	Hispanic (n = 455,356)	Non- Hispanic Asian(n = 32,499)	Non- Hispanic Black or African American (n = 22,903)	Non- Hispanic Native Hawaiian and Pacific Islander (n = 13,115)	Non- Hispanic American Indian or Alaska Native (n = 10,424)	Multiple Races (n = 81,134)
	%	%	%	%	%	%	%
**Sex**							
Female	50.5	50.4	55.4	43.0	45.4	51.8	51.9
Male	49.5	49.6	44.6	57.0	54.6	48.2	48.1
**Age (median, IQR)**	29, 14–45	29, 14–44	37, 20–53	28, 14–44	26, 8–40	30, 12–51	31, 17–46
**Age**							
< 18	30.4	31.5	22.7	31.3	38.7	33.8	25.4
18 to 29	19.8	20.1	13.7	22.3	17.8	15.5	20.6
30 to 39	16.8	16.3	18.7	15.8	18.3	13.3	19.1
40 to 49	13.5	13.5	15.2	11.8	12.1	11.1	14.2
50 to 59	9.4	9.3	13.0	9.0	7.8	11.0	8.8
60 to 69	6.3	6.0	8.9	6.6	3.9	9.4	6.6
70 to 79	2.6	2.2	5.4	2.3	1.1	4.0	3.7
80 to 110	1.2	1.1	2.4	0.9	0.3	1.9	1.6
**Born outside the United States**							
No	95.9	68.4	33.8	60.4	70.5	87.8	79.8
Yes	1.8	26.1	57.8	23.6	20.0	1.1	15.3
Unknown	2.3	5.5	8.4	16.0	9.6	11.1	4.9
**Education (county-level)**							
< 7% had high school degree	19.8	13.0	19.1	11.3	14.4	6.1	15.6
7 to < 10% had high school degree	21.7	17.0	20.9	15.8	16.4	17.6	19.4
10 to < 15% had high school degree	29.9	33.8	31.2	37.2	31.6	16.1	31.5
15%+ had high school degree	21.6	30.4	26.5	32.2	34.1	50.9	28.6
Unknown	7.1	5.7	2.3	3.5	3.5	9.3	4.9
**Median household income (county-level)**							
< 60,0000	16.3	25.7	22.6	33.6	27.4	63.6	24.8
60,000 to < 80,000	24.9	29.0	23.9	27.6	30.4	13.9	26.6
80,000 to < 100,000	22.8	19.5	21.0	16.8	19.2	7.3	20.4
100,000+	29.1	20.1	30.2	18.9	19.5	6.0	23.5
Unknown	7.0	5.7	2.3	3.2	3.5	9.3	4.8
**Baseline Charlson Comorbidity Index**							
0	87.3	87.2	85.7	84.9	87.7	84.2	82.6
1	7.8	7.9	8.4	8.6	7.3	9.0	9.6
2+	4.9	4.9	5.9	6.4	5.0	6.7	7.8
**Obesity before baseline**							
No	79.0	76.6	90.7	78.9	67.2	80.9	72.1
Yes	21.0	23.4	9.3	21.1	32.8	19.1	27.9

**Table 2:** Odds ratio of COVID-19 related outcome among race and ethnicity minority compared to non-Hispanic whites in Utah

	COVID-19 related outcome				Model 1	Model 2	Model 3
	Yes		No		adjusted for birthplace	additionally adjusted for SES and CCI	additionally adjusted for obesity
	n	(%)	n	(%)	OR (95% CI)^a^	OR (95% CI)^b^	OR (95% CI)^c^
**Hospitalization with COVID-19 diagnosis**							
Non-Hispanic White	7,301	1.2	608,130	98.8	Referent	Referent	Referent
Hispanic	8,343	1.8	447,013	98.2	1.53 (1.47, 1.60)	1.46 (1.39, 1.52)	1.42 (1.36, 1.49)
Asian	385	1.2	32,114	98.8	0.74 (0.64, 0.86)	0.75 (0.65, 0.88)	0.86 (0.74, 1.01)
Black or African American	328	1.4	22,575	98.6	1.11 (0.95, 1.31)	1.01 (0.85, 1.20)	1.02 (0.85, 1.22)
NHPI	467	3.6	12,648	96.4	4.34 (3.49, 5.39)	4.08 (3.26, 5.10)	3.39 (2.69, 4.25)
American Indian or Alaska Native	307	2.9	10,117	97.1	2.20 (1.79, 2.69)	2.03 (1.64, 2.52)	2.10 (1.69, 2.61)
Multiple Races	2,185	2.7	78,949	97.3	2.03 (1.88, 2.19)	1.78 (1.65, 1.93)	1.71 (1.58, 1.86)
**ICU with COVID-19 diagnosis**							
Non-Hispanic White	2,414	0.4	613,017	99.6	Referent	Referent	Referent
Hispanic	2,913	0.6	452,443	99.4	1.58 (1.47, 1.69)	1.51 (1.40, 1.63)	1.49 (1.37, 1.61)
Asian	136	0.4	32,363	99.6	0.81 (0.63, 1.03)	0.86 (0.66, 1.12)	0.98 (0.75, 1.28)
Black or African American	114	0.5	22,789	99.5	1.30 (0.98, 1.74)	1.16 (0.85, 1.58)	1.18 (0.86, 1.63)
NHPI	176	1.3	12,939	98.7	4.60 (3.21, 6.60)	4.17 (2.87, 6.08)	3.44 (2.35, 5.03)
American Indian or Alaska Native	145	1.4	10,279	98.6	2.72 (1.98, 3.74)	2.49 (1.78, 3.48)	2.61 (1.85, 3.68)
Multiple Races	844	1.0	80,290	99.0	2.46 (2.16, 2.80)	2.07 (1.80, 2.38)	1.94 (1.68, 2.24)
**All-cause death**							
Non-Hispanic White	5,829	0.9	609,602	99.1	Referent	Referent	Referent
Hispanic	4,362	1.0	450,994	99.0	1.37 (1.30, 1.45)	1.27 (1.20, 1.35)	1.27 (1.20, 1.35)
Asian	362	1.1	32,137	98.9	0.97 (0.83, 1.14)	0.99 (0.83, 1.18)	1.00 (0.84, 1.18)
Black or African American	239	1.0	22,664	99.0	1.66 (1.35, 2.06)	1.39 (1.10, 1.74)	1.39 (1.10, 1.75)
NHPI	215	1.6	12,900	98.4	4.70 (3.46, 6.37)	4.13 (3.00, 5.68)	4.10 (2.98, 5.65)
American Indian or Alaska Native	211	2.0	10,213	98.0	2.04 (1.59, 2.61)	1.87 (1.43, 2.44)	1.87 (1.43, 2.44)
Multiple Races	1,443	1.8	79,691	98.2	1.89 (1.72, 2.07)	1.64 (1.48, 1.81)	1.64 (1.48, 1.81)
**COVID-19-specific death**							
Non-Hispanic White	400	0.1	615,031	99.9	Referent	Referent	Referent
Hispanic	608	0.1	454,748	99.9	2.10 (1.75, 2.53)	2.20 (1.81, 2.68)	2.20 (1.80, 2.68)
Asian	57	0.2	32,442	99.8	1.50 (0.93, 2.44)	1.23 (0.74, 2.04)	1.32 (0.79, 2.20)
Black or African American	22	0.1	22,881	99.9	1.38 (0.71, 2.65)	1.06 (0.52, 2.13)	1.17 (0.57, 2.38)
NHPI	49	0.4	13,066	99.6	10.74 (3.85, 29.96)	11.28 (3.97, 32.06)	10.16 (3.57, 28.91)
American Indian or Alaska Native	45	0.4	10,379	99.6	6.80 (3.03, 15.25)	6.15 (2.67, 14.16)	6.77 (2.94, 15.63)
Multiple Races	183	0.2	80,951	99.8	2.81 (2.08, 3.81)	2.50 (1.82, 3.44)	2.41 (1.74, 3.32)

Abbreviation: SES, socioeconomic status; OR, odds ratio; CI, confidence interval; CCI, Charlson Comorbidity Index; NHPI, Native Hawaiian and Pacific Islander

a. Adjusted for sex, age, and birthplace

b. Additionally adjusted for county-level education, county-level income, and baseline Charlson Comorbidity Index

c. Additionally adjusted for obesity before baseline

**Table 3:** Odds ratio of COVID-19 related outcome among race and ethnicity minority compared to Non-Hispanic Whites in Utah, stratified by sex

	Had COVID-19 related outcome		OR (95% CI)^a^	Had COVID-19 related outcome		OR (95% CI)^a^	P for interaction between race/ethnicity and sex
	n	(%)		n	(%)		
	Male			Female			
Hospitalization with COVID-19 diagnosis							
Non-Hispanic White	3,488	1.1	Referent	3,813	1.2	Referent	
Hispanic	3,933	1.7	1.41 (1.31, 1.50)	4,410	1.9	1.43 (1.35, 1.52)	0.594
Asian	174	1.2	0.81 (0.64, 1.02)	211	1.2	0.92 (0.74, 1.13)	0.748
Black or African American	188	1.4	1.13 (0.89, 1.43)	140	1.4	0.89 (0.68, 1.17)	0.164
NHPI	257	3.6	4.27 (3.05, 5.98)	210	3.5	2.66 (1.95, 3.64)	0.030
American Indian or Alaska Native	131	2.6	1.93 (1.39, 2.67)	176	3.3	2.25 (1.68, 3.02)	0.439
Multiple Races	982	2.5	1.59 (1.41, 1.79)	1,203	2.9	1.83 (1.64, 2.05)	0.118
**ICU with COVID-19 diagnosis**							
Non-Hispanic White	1,299	0.4	Referent	1,115	0.4	Referent	
Hispanic	1,617	0.7	1.57 (1.41, 1.74)	1,296	0.6	1.38 (1.23, 1.56)	0.020
Asian	73	0.5	0.90 (0.63, 1.30)	63	0.4	1.10 (0.73, 1.64)	0.738
Black or African American	82	0.6	1.40 (0.96, 2.06)	32	0.3	0.77 (0.43, 1.37)	0.091
NHPI	102	1.4	3.94 (2.37, 6.57)	74	1.2	2.85 (1.61, 5.07)	0.377
American Indian or Alaska Native	64	1.3	1.95 (1.22, 3.11)	81	1.5	3.64 (2.16, 6.13)	0.100
Multiple Races	422	1.1	1.89 (1.55, 2.30)	422	1.0	2.01 (1.64, 2.47)	0.697
**All-cause death**							
Non-Hispanic White	3,098	1.0	Referent	2,731	0.9	Referent	
Hispanic	2,374	1.1	1.28 (1.19, 1.39)	1,988	0.9	1.26 (1.15, 1.38)	0.693
Asian	199	1.4	1.08 (0.85, 1.36)	163	0.9	0.93 (0.71, 1.21)	0.526
Black or African American	160	1.2	1.40 (1.07, 1.84)	79	0.8	1.37 (0.90, 2.09)	0.935
NHPI	127	1.8	4.29 (2.87, 6.40)	88	1.5	3.69 (2.19, 6.23)	0.635
American Indian or Alaska Native	121	2.4	2.42 (1.67, 3.50)	90	1.7	1.39 (0.93, 2.07)	0.022
Multiple Races	798	2.0	1.70 (1.49, 1.94)	645	1.5	1.56 (1.33, 1.82)	0.579
**COVID-19-specific death**							
Non-Hispanic White	255	0.1	Referent	145	0.0	Referent	
Hispanic	378	0.2	2.21 (1.71, 2.85)	230	0.1	2.20 (1.58, 3.06)	0.745
Asian	35	0.2	1.39 (0.75, 2.57)	22	0.1	1.33 (0.51, 3.45)	0.653
Black or African American	14	0.1	1.22 (0.52, 2.85)	*	*	1.13 (0.30, 4.21)	0.939
NHPI	29	0.4	13.10 (3.06, 56.08)	20	0.3	6.95 (1.45, 33.26)	0.627
American Indian or Alaska Native	24	0.5	4.65 (1.72, 12.57)	21	0.4	24.04 (3.00, 192.41)	0.240
Multiple Races	113	0.3	2.47 (1.66, 3.68)	70	0.2	2.37 (1.33, 4.24)	0.774

Abbreviation: OR, odds ratio; CI, confidence interval; NHPI, Native Hawaiian and Pacific Islander

a. Adjusted for age, birthplace, county-level education, county-level income, obesity, and baseline Charlson Comorbidity Index

* Cell counts < 11 were suppressed in accordance with IRB policy to protect patient privacy

**Table 4:** Odds ratio of COVID-19 related outcome among race and ethnicity minority compared to Non-Hispanic Whites in Utah, stratified by obesity before baseline

	Had COVID-19 related outcome		OR (95% CI)^a^	Had COVID-19 related outcome		OR (95% CI)^a^	P for interaction between race/ethnicity and obesity
	n	(%)		n	(%)		
	Obesity before baseline			No obesity before baseline			
**Hospitalization with COVID-19 diagnosis**							
Non-Hispanic White	3,686	2.8	Referent	3,615	0.7	Referent	
Hispanic	4,210	4.0	1.38 (1.31, 1.45)	4,133	1.2	1.43 (1.36, 1.50)	0.303
Asian	79	2.6	0.74 (0.59, 0.94)	306	1.0	0.76 (0.67, 0.87)	0.180
Black or African American	148	3.1	0.94 (0.79, 1.11)	180	1.0	1.15 (0.99, 1.34)	0.095
NHPI	330	7.7	2.97 (2.62, 3.35)	137	1.6	2.56 (2.15, 3.05)	0.025
American Indian or Alaska Native	143	7.2	2.22 (1.86, 2.66)	164	1.9	1.99 (1.69, 2.34)	0.554
Multiple Races	1,317	5.8	1.95 (1.82, 2.09)	868	1.5	1.59 (1.47, 1.72)	0.000
**ICU with COVID-19 diagnosis**							
Non-Hispanic White	1,292	1.0	Referent	1,122	0.2	Referent	
Hispanic	1,496	1.4	1.45 (1.34, 1.58)	1,417	0.4	1.56 (1.43, 1.71)	0.073
Asian	34	1.1	0.94 (0.66, 1.34)	102	0.3	0.79 (0.64, 0.98)	0.951
Black or African American	54	1.1	1.00 (0.75, 1.31)	60	0.3	1.18 (0.90, 1.53)	0.318
NHPI	131	3.0	3.40 (2.81, 4.12)	45	0.5	2.78 (2.05, 3.77)	0.150
American Indian or Alaska Native	76	3.8	3.25 (2.55, 4.15)	69	0.8	2.45 (1.90, 3.15)	0.233
Multiple Races	547	2.4	2.27 (2.04, 2.52)	297	0.5	1.68 (1.47, 1.92)	0.002
**All-cause death**							
Non-Hispanic White	2,373	1.8	Referent	3,456	0.7	Referent	
Hispanic	1,871	1.8	1.30 (1.21, 1.39)	2,491	0.7	1.21 (1.14, 1.29)	0.011
Asian	52	1.7	1.00 (0.74, 1.34)	310	1.1	1.01 (0.88, 1.15)	0.887
Black or African American	77	1.6	1.00 (0.79, 1.27)	162	0.9	1.46 (1.23, 1.73)	0.014
NHPI	136	3.2	3.56 (2.94, 4.32)	79	0.9	3.26 (2.57, 4.14)	0.183
American Indian or Alaska Native	72	3.6	1.81 (1.40, 2.34)	139	1.6	1.55 (1.28, 1.86)	0.497
Multiple Races	636	2.8	1.70 (1.55, 1.88)	807	1.4	1.66 (1.53, 1.81)	0.389
**COVID-19-specific death**							
Non-Hispanic White	212	0.2	Referent	188	0.0	Referent	
Hispanic	291	0.3	1.95 (1.60, 2.36)	317	0.1	2.19 (1.79, 2.69)	0.274
Asian	12	0.4	2.04 (1.12, 3.72)	45	0.2	1.66 (1.16, 2.37)	0.982
Black or African American	*	*	0.70 (0.29, 1.70)	17	0.1	2.48 (1.50, 4.10)	0.018
NHPI	32	0.7	6.40 (4.30, 9.51)	17	0.2	8.78 (5.21, 14.79)	0.467
American Indian or Alaska Native	16	0.8	4.64 (2.75, 7.83)	29	0.3	5.84 (3.86, 8.84)	0.391
Multiple Races	103	0.5	2.74 (2.14, 3.50)	80	0.1	2.39 (1.82, 3.14)	0.666

Abbreviation: OR, odds ratio; CI, confidence interval; NHPI, Native Hawaiian and Pacific Islander

a. Adjusted for sex, age, birthplace, county-level education, county-level income, and baseline Charlson Comorbidity Index

* Cell counts < 11 were suppressed in accordance with IRB policy to protect patient privacy

## Data Availability

In order to protect patient privacy and comply with relevant regulations, identified data are unavailable. Requests for deidentified data from qualified researchers with appropriate ethics board approvals and relevant data use agreements will be processed by the Intermountain Office of Research, officeofresearch@imail.org
